# Symptomatic diagnoses in primary care: an observational cohort study

**DOI:** 10.3399/BJGPO.2023.0234

**Published:** 2024-12-18

**Authors:** Mika T Lehto, Timo Kauppila, Hannu Kautiainen, Merja K Laine, Ossi Rahkonen, Kaisu H Pitkälä

**Affiliations:** 1 Department of General Practice and Primary Health Care, University of Helsinki and Helsinki University Hospital, Helsinki, Finland; 2 Vantaa Health Centre, Vantaa, Finland; 3 MedCare Ltd, Äänekoski, Finland; 4 Folkhälsan Research Center, Helsinki, Finland; 5 Department of Public Health, University of Helsinki, Helsinki, Finland

**Keywords:** Diagnosis, Primary healthcare, Symptomatic, General practitioners

## Abstract

**Background:**

It can be impossible to assign a definitive diagnosis for symptoms reported or observed by primary health care patients. In these situations, symptomatic diagnoses are often used.

**Aim:**

The aim of the present study was to examine the proportion of symptomatic diagnoses among primary health care patients. We also explored which symptomatic diagnoses were most frequently recorded, as well as their distribution by age and sex.

**Design & setting:**

This is a register-based study carried out in the public primary health care service of the city of Vantaa, Finland.

**Method:**

Diagnoses were entered according to the 10th revision of the International Classification of Diseases (ICD-10). The data consisted of every diagnosis entered into the electronic health record between 1 January 2016 and 31 December 2018. Both absolute numbers and relative proportions of various symptomatic diagnosis recordings (chapter ‘R’) were reported.

**Results:**

Of all the recorded diagnoses (*n* = 503 001), the proportion of R-diagnoses was 13.5% (*n* = 67 905). Diagnoses of symptoms and signs involving the digestive system and abdomen (R10–19) (3.7% of all; *n* = 18 550), the circulatory and respiratory systems (R00–09) (3.5%; *n* = 17 426), general symptoms and signs (R50–69) (3.0%; *n* = 15 165), and the skin and subcutaneous tissue (R20–23) (2.0%; *n* = 9812) were most prevalent. Age was also a major factor determining how symptomatic diagnoses were distributed between men and women. Overall, symptomatic diagnoses were more common among women than men (14.1% and 12.4%, respectively). The major categories of symptoms and signs involving the digestive system and abdomen, the skin and the subcutaneous tissue, and general symptoms and signs, were more predominant among women, while symptoms and signs involving the circulatory and respiratory systems were more common among men.

**Conclusion:**

A symptomatic diagnosis code was recorded in about one eighth of GP appointments, although there were significant sex differences in the prevalence within and between diagnosis groups.

## How this fits in

The underlying reasons for the symptoms presented by patients to primary health care cannot always be explained by a medical diagnosis. However, little is known about the extent to which various symptom-based diagnoses are used as reasons for visits, and how gender and age influence these diagnoses. Approximately one in eight diagnoses in primary health care is symptomatic, indicating the uncertainty regarding the underlying diagnosis.

## Introduction

In primary health care, GPs deal with patients during the onset of diseases and time is required to assess whether an actual disease will gradually begin to declare itself, or whether the problem will eventually turn out to be self-limiting.^1^ Consequently, GPs often need symptomatic diagnoses to describe the reasons for consultations or visits. Patients seek GPs’ help at the early stages of illness and may also present with multiple symptoms that cannot be fully explained by an identified medical condition.^2,3^ Therefore, it is sometimes impossible to assign a definitive diagnosis as the cause of reported or observed symptoms.^2,3^

However, some characterisation of the cause for patients consulting GPs is essential for administrative reasons,^4–7^ educational reasons,^8^ cues for follow-up, and for improving the quality of care.^4,6,7^ Precise diagnoses cannot always be assigned, but as classification of the reason to meet with a physician is required, diagnostic classifications also contain symptom-based diagnoses, for example diagnoses which describe only the symptoms, signs and abnormal clinical findings but do not suggest any specific illnesses behind these symptoms.^2,3,9,10^ In the 10^th^ revision of the International Classification of Diseases (ICD-10) these symptom-based diagnoses constitute the chapter ‘R’.^11^

There are a few reports concerning the epidemiology of symptom-based diagnoses in primary health care. A Finnish study using the ninth revision of the International Classification of Diseases (ICD-9) found that symptomatic diagnoses made up 13% of all recorded diagnoses.^9^ However, in another Finnish study using ICD-10 at an accuracy of four characters (including subcategories), chapter R diagnoses comprised 3% of the most frequently recorded diagnoses.^10^ According to the Finnish nationwide care register for primary health care (AvoHilmo), the percentage of symptomatic diagnoses during 2022 was 12%. Thus, these data vary according to setting, time, and reasons for collection. There are differences in health behaviour and diagnoses of chronic diseases between men and women, as well as how men and women seek medical help.^12^ However, little is known about how men and women exhibit various symptoms and how age modifies this.

The aim of the present study was to explore the proportion of symptomatic diagnoses for all visit reasons in a cohort of primary health care patients, and to examine which symptom-based diagnoses were most frequently recorded between 2016 and 2018. We also explored the sex and age distributions of the most common symptomatic diagnoses.

## Method

This study was performed in the city of Vantaa, Finland. With a population of 228 678 (31 January 2019), Vantaa is the fourth most populated city in Finland. The present work is a register-based study in the public primary health care service of Vantaa. Data were gathered from the electronic health record (EHR) system used in the primary health care service in Vantaa and consisted of every diagnosis entered into the EHR between 1 January 2016 and 31 December 2018. In Finland, primary health care is non-profit-making and municipalities, which fund this activity mostly with tax income, maintain it as well as the EHR systems. GPs are employed and directly governed by the municipal health administration.

Data from the Vantaa health centre were obtained from the Graphic Finstar-EHR system (GFS). Information regarding the diagnosis is entered into the GFS system as structural data based on the ICD-10 classification system. The ICD-10 diagnoses were entered during the patients’ visits to GPs. The system assisted the GP in finding the correct diagnosis code or allowed the GP to enter the desired code for the diagnosis directly. The diagnosis code was always chosen and thereby decided by the GP. The GP’s input was to give at least the three first letters and/or numbers of their suggestion as a diagnosis. The system then displayed a table of diagnoses which contain those cues originated by the GP, who was then able to choose the diagnosis code they considered most appropriate. As a result of the use of electronic reminders, the diagnosis recording rate was over 90% in Vantaa’s primary health care service during the study period.^13^

The main outcome in the present study was the proportion of the diagnosis codes in chapter ‘R’ based on ICD-10 classification for all reasons for visits, when entered as a primary diagnosis code in primary health care GP visits. Chapter R of the ICD-10 contains symptoms, signs, and abnormal clinical and laboratory findings that are not classified elsewhere.^11^ We focused on those R-diagnoses which described only symptoms and signs of abnormal clinical findings (R00–R69). To obtain sufficient data to run statistical analyses we grouped these recorded R-diagnoses in eight blocks in accordance with the ICD-10 as follows: R00–R09 (symptoms and signs involving the circulatory and respiratory systems), R10–R19 (symptoms and signs involving the digestive system and abdomen), R20–R23 (symptoms and signs involving the skin and subcutaneous tissue), R25–R29 (symptoms and signs involving the nervous and musculoskeletal systems), R30–R39 (symptoms and signs involving the urinary system), R40–R46 (symptoms and signs involving cognition, perception, emotional state, and behaviour), R47–R49 (symptoms and signs involving speech and voice), and R50–R69 (general symptoms and signs). The age and sex of the patients was also retrieved from the EHR system.

All visits which did not have a diagnosis code entered were excluded from the analysis and the proportions of R-diagnoses were calculated using the total amount of diagnosis-included visits as a denominator.

### Statistical analysis

The descriptive statistics were presented as means with standard deviations (SDs) or as counts with percentages. The rates (per 100 visits) and relative rate (RR) between sexes derived from a 4-knot restricted cubic splines random-effects Poisson models with four knots at the fifth, 35^th^, 65^th^, and 95^th^ percentiles. Knot locations were based on Harrell’s recommended percentiles.^14^ All analyses were performed using STATA software (version 17.0).

## Results

A total of 305 650 different visits with diagnoses among women, and 197 351 among men (total 503 001) were included in the study. The mean age of women was 47 years (SD 24) and men 44 years (SD 26). Symptomatic diagnoses made up 13.5% (*n* = 67 905) of all diagnoses. The proportion of visits with symptom-based diagnoses recorded among women and men was 14.1% (*n* = 42 982) and 12.4% (*n* = 24 468), respectively.

The four most frequently recorded diagnosis groups were symptoms and signs involving the digestive system and abdomen (R10–19) (3.7% of all diagnoses; *n* = 18 550), symptoms and signs involving the circulatory and respiratory systems (R00–09) (3.5% of all diagnoses; *n* = 17 426), general symptoms and signs (R50–69) (3.0% of all diagnoses; *n* = 15 165), and symptoms and signs involving the skin and subcutaneous tissue (R20–23) (2.0% of all diagnoses; *n* = 9812) (Table 1). The most common individual symptomatic diagnoses were R10 (abdominal and pelvic pain), R05 (cough), R23 (other skin changes), R07 (pain in throat and chest), R06 (abnormalities in breathing), R53 (malaise and fatigue), R22 (localised swelling, mass, and lump of skin and subcutaneous tissue), and R51 (headache) (Table 1).

**Table 1.** Rates, percentages, and cumulative percentages in each R category of symptoms and signs involving (R10–19) the digestive system and abdomen, (R00–09) the circulatory and respiratory systems of all recorded diagnoses, (R50–69) general symptoms and signs and (R20–23) those involving the skin and subcutaneous tissue

ICD-10 code	Description	*n*	Percentage (%)	Cumulative percentage (%)
R10–19	All (symptoms and signs involving the digestive system and abdomen)	18 550		
R10	Abdominal and pelvic pain	15 299	82.5	82.5
R11	Nausea and vomiting	984	5.3	87.8
R12	Heartburn	1159	6.3	94.0
R13	Dysphagia	446	2.4	96.4
R14	Flatulence and related conditions	115	0.6	97.1
R15	Faecal incontinence	96	0.5	97.6
R16	Hepatomegaly and splenomegaly, not elsewhere classified	7	0.0	97.6
R17	Unspecified jaundice	35	0.2	97.8
R18	Ascites	40	0.2	98.0
R19	Other symptoms and signs involving the digestive system and abdomen	369	2.0	100.0
R00–09	All (symptoms and signs involving the circulatory and respiratory systems)	17 426		
R00	Abnormalities of heartbeat	975	5.6	5.6
R01	Cardiac murmurs and other cardiac sounds	388	2.2	7.8
R02	Gangrene, not elsewhere classified	1	0.1	7.8
R03	Abnormal blood-pressure reading, without diagnosis	484	2.8	10.6
R04	Haemorrhage from respiratory passages	838	4.8	15.4
R05	Cough	6119	35.1	50.5
R06	Abnormalities of breathing	3587	20.6	71.1
R07	Pain in throat and chest	4886	28.0	99.2
R09	Other symptoms and signs involving the the circulatory and respiratory systems	148	0.9	100.00
R50-69	All (general symptoms and signs)	15 165		
R50	Fever of other and unknown origin	1875	12.4	12.4
R51	Headache	3168	20.9	33.3
R52	Pain, not elsewhere classified	3122	20.6	53.8
R53	Malaise and fatigue	3281	21.6	75.5
R54	Senility	11	0.0	75.6
R55	Syncope and collapse	441	2.9	78.5
R56	Convulsions, not elsewhere classified	76	0.5	79.0
R57	Shock, not elsewhere classified	0		
R58	Haemorrhage, not elsewhere classified	29	0.2	79.2
R59	Enlarged lymph nodes	440	2.9	82.1
R60	Oedema, not elsewhere classified	1907	12.6	94.6
R61	Hyperhidrosis	147	1.0	95.6
R62	Lack of expected normal physiological development	53	0.4	95.9
R63	Symptoms and signs concerning food and fluid intake	469	3.1	99.0
R64	Cachexia	3	0.0	99.1
R65	Systemic inflammatory response syndrome	0		
R68	Other general symptoms and signs	141	0.9	100.0
R69	Unknown and unspecified causes of morbidity	2	0.0	100.0
R20–23	All (symptoms and signs involving the skin and subcutaneous tissue)	9812		
R20	Disturbances of skin sensation	495	5.0	5.0
R21	Rash and other non-specific skin eruption	1164	11.9	16.9
R22	Localised swelling, mass, and lump of skin and subcutaneous tissue	3235	33.0	49.9
R23	Other skin changes	4918	50.1	100.00

ICD-10 = International Classification of Diseases 10th Revision.

**Figure 1.**
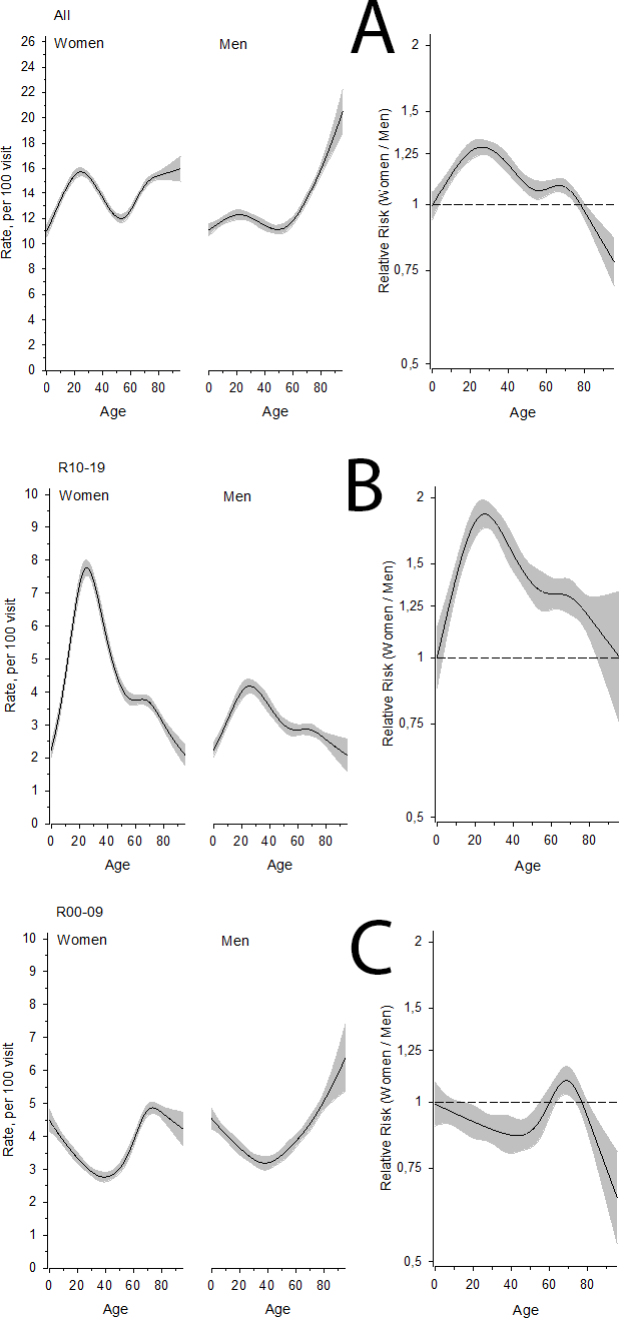
Panel A: the rate of symptomatic diagnoses as a function of age in different sexes. Panel B: the rate of symptoms and signs involving the digestive system and abdomen as a function of age in different sexes. Panel C: the rate of symptoms and signs involving the circulatory and respiratory systems as a function of age in different sexes. In the right panels, the risk ratio (RR, women/men) is shown as a function of age. Mean (line) and 95% confidence intervals (shaded area) are shown. In all panels mean (line) and 95% confidence intervals (shaded area) are shown. See Figure 2 for Panel D and E.

**Figure 2.**
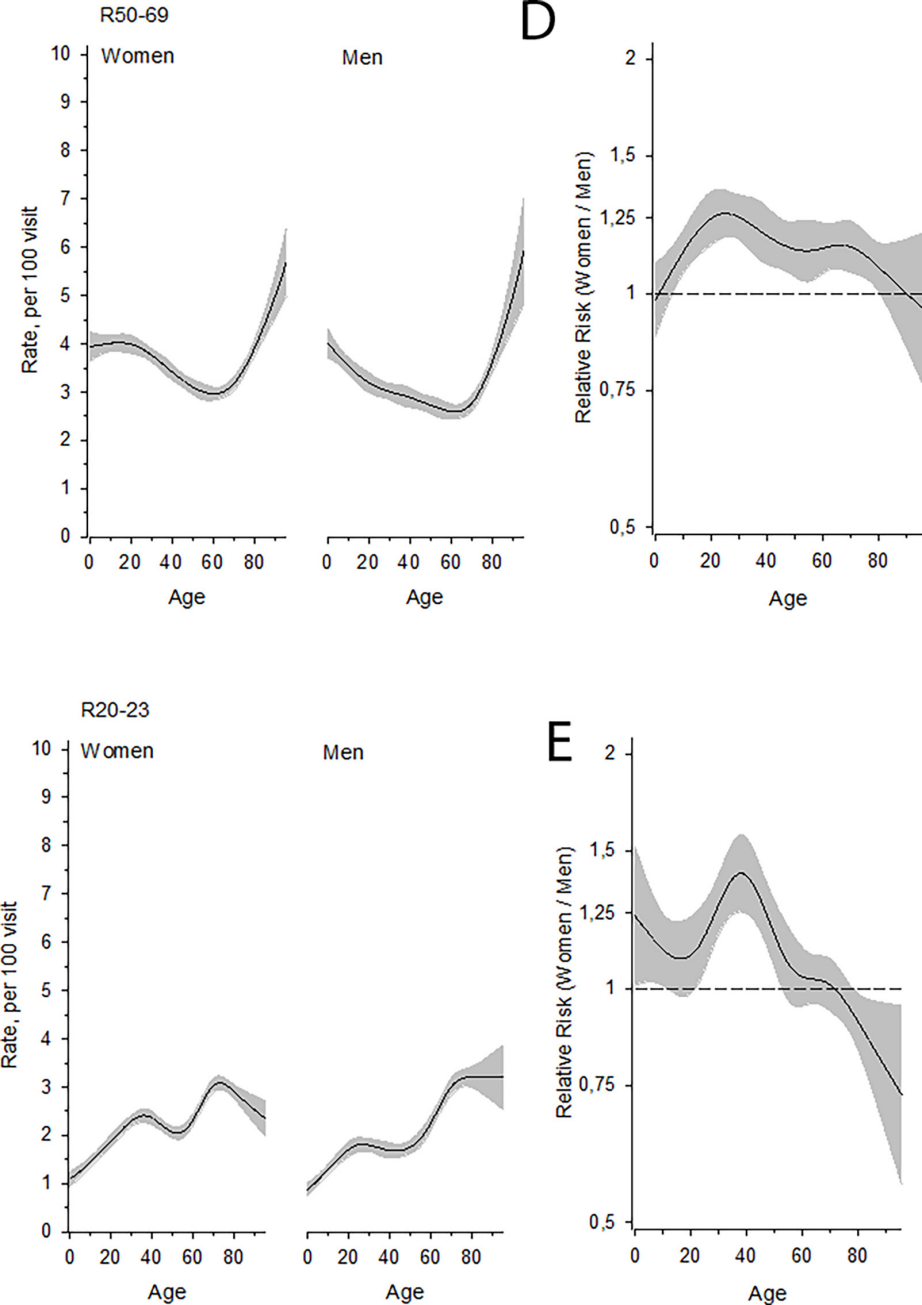
Panel D: the rate of general symptoms and signs as a function of age in different sexes. Panel E: the rate of symptoms and signs involving the skin and subcutaneous tissue as a function of age in different sexes. In the right panels, the risk ratio (RR, women/men) is shown as a function of age. Mean (line) and 95% confidence intervals (shaded area) are shown. In all panels mean (line) and 95% confidence intervals (shaded area) are shown.

Symptom-based diagnoses were recorded more often among women than men (age-adjusted RR 1.12, 95% confidence intervals [95% CI] 1.10 to 1.14) (Table 2). The probability of having a recorded symptom-based diagnosis increased among men with increasing age, especially after the age of 50 years, and became more common among men than women at around the age of 80 years (Figure 1, Panel A). The majority of symptomatic diagnoses increased with ageing, but many diagnoses also had peaks in childhood and young adulthood (Figure 1, Panels A–C, Figure 2 Panels D–E). Recorded diagnoses in diagnosis groups R10–19 (symptoms and signs involving the digestive system and abdomen) were more common among women than men in all age groups (Figure 1, Panel B). Recorded diagnoses in diagnosis groups R00–09 (symptoms and signs involving the circulatory and respiratory systems) were more common among women than men in all age groups except among 60–80-year-olds (Figure 1, Panel C). R50–69 (general symptoms and signs) were more common among women than among men in nearly all age groups (Figure 2, Panel D). R20–23 (symptoms and signs involving the skin and subcutaneous tissue) were more common among women than men until the age of 70 years, after which men outnumbered women (Figure 2, Panel E).

**Table 2.** Symptom-based diagnosis recording rates per 100 visits. Mean and 95% confidence intervals are presented

ICD-10 code	Description	Total rate (95% CI)	Rate for women (95% CI)	Rate for men (95% CI)	Rr^a^ (95% CI)
All R-groups	Symptoms, signs and abnormal clinical and laboratory findings, not elsewhere classified	13.5 (13.4 to 13.6)	14.1 (14.0 to 14.2)	12.5 (12.4 to 12.7)	1.12 (1.10 to 1.14)
R00–09	Symptoms and signs involving the circulatory and respiratory systems	3.9 (3.8 to 4.0)	3.8 (3.7 to 3.9)	4.0 (3.9 to 4.1)	0.93 (0.91 to 0.96)
R10–19	Symptoms and signs involving the digestive system and abdomen	4.1 (4.0 to 4.2)	4.7 (4.6 to 4.8)	3.1 (3.0 to 3.2)	1.55 (1.51 to 1.60)
R20–23	Symptoms and signs involving the skin and subcutaneous tissue	2.2 (2.1 to 2.6)	2.3 (2.2 to 2.4)	2.1 (2.0 to 2.2)	1.10 (1.05 to 1.14)
R25–29	Symptoms and signs involving the nervous and musculoskeletal systems	0.1 (0.0 to 0.2)	0.1 (0.0 to 0.2)	0.1 (0.0 to 0.2)	1.00 (0.99 to 1.01)
R30–39	Symptoms and signs involving the urinary system	0.5 (0.4 to 0.6)	0.4 (0.3 to 0.5)	0.7 (0.6 to 0.8)	0.49 (0.45 to 0.53)
R40–46	Symptoms and signs involving cognition, perception, emotional state and behaviour	0.8 (0.7 to 0.9)	0.9 (0.8 to 1.0)	0.7 (0.6 to 0.8)	1.29 (1.20 to 1.38)
R47–49	Symptoms and signs involving speech and voice	0.1 (0.0 to 0.2)	0.1 (0.0 to 0.2)	0.0 (0.0 to 0.1)	1.18 (0.90 to 1.53)
R50–69	General symptoms and signs	3.4 (3.3 to 3.5)	3.6 (3.5 to 3.7)	3.1 (3.0 to 3.2)	1.14 (1.11 to 1.18)

a Age adjusted. CI = confidence intervals. RR = risk ratio.

Table 2 shows the proportion of men and women having various symptomatic diagnoses and RR for women compared to men. Recorded diagnoses in diagnosis groups R10–19 (symptoms and signs involving the digestive system and abdomen) (Figure 1, Panel B), R20–23 (symptoms and signs involving the skin and subcutaneous tissue) (Figure 2, Panel E), R40–46 (symptoms and signs involving cognition, perception, emotional state and behaviour), and R50–69 (general symptoms and signs) (Figure 2, Panel D) were more common among women than men. Recorded diagnoses in diagnosis groups R00–09 (symptoms and signs involving the circulatory and respiratory systems) (Figure 1, Panel C) and R30–39 (symptoms and signs involving the urinary system) were more common among men than women. However, at the age of around 70 years the frequency of R00–09 diagnoses peaked among women (Figure 1, Panel C).

## Discussion

### Summary

Symptom and sign-based diagnoses represented 13.5% of all recorded diagnoses in a primary health care setting. Symptoms and signs involving the digestive system and abdomen (R10–19), the circulatory and respiratory systems (R00–09), general symptoms and signs (R50–69), and symptoms and signs involving the skin and subcutaneous tissue (R20–23) were the most common symptom-based diagnosis groups. Generally, symptom-based diagnoses were more common among women than men. However, there were several sex differences in the prevalence within and between the diagnosis groups. In addition, age was also a determinant for the sex distribution in diagnosis groups. The most common individual symptom-based diagnoses were R10 (abdominal and pelvic pain), R05 (cough), R23 (other skin changes), R07 (pain in throat and chest), and R06 (abnormalities in breathing).

### Strengths and limitations

A major strength of this study was the completeness of the data, as well as the relatively large number of GPs and patients who were included. The data are representative of patients in the primary health care setting. The study design ensured that the symptoms stated and explored were dealt with by the GPs.

The prevalence of symptoms managed may have been underestimated for various reasons. First, GPs mostly recorded only one diagnostic label for each patient, although in about one third of all primary health care encounters, patients present with several problems during the same consultation or visit.^15^ Second, the present study is based on the ICD-10 system which has been criticised for being too stiff^16^ and unreliable^17^ for use in primary health care. The ICD system has been considered too specific to describe problems relevant to the work of GPs. It would be inaccurate to state that patients are usually seeking primary health care because of a particular diagnosis with which they need to consult a GP. Rather, an encounter in general practice often starts with a patient presenting to their primary care provider with one or more symptoms or complaints that constitutes a statement on why they visit the doctor in the first place. As the ICD system is primarily focused on diagnoses, albeit using the R-diagnoses, it does not take into account the worries, concerns, and fears that contribute to the patient’s decision to contact health care. Third, we cannot rule out that some GPs tend to place more emphasis on diseases than symptoms. Thus, when observing a pattern of some particular symptoms, some GPs may automatically use specific diagnosis codes. A number of factors may impact the results in this study, in particular, underlying comorbidities, especially when age is taken into account. Unfortunately, the available data did not allow us to explore the potential association between symptomatic diagnoses and recorded chronic diseases.

As the main outcome in the present study was the proportion of R-diagnoses among all recorded visits that included a diagnosis, the study did not take into account symptom-based diagnoses on a patient-by-patient level. Therefore, the study cannot explore the extent to which the same patient consults a GP with the same complaint on more than one occasion.

A further limitation is that even though the rate of recording a diagnosis during the study period exceeded 90%, almost 10% of patients did not receive a diagnosis during their GP visit. It is likely that at least one underlying reason not to record a diagnosis altogether is the difficulty of recognising a specific disease that is causing the presenting complaint. Therefore, the proportion of symptom-based diagnoses is probably an underestimate. Furthermore, GPs’ diagnostic labelling was not validated. A high GP inter-rater variability may have been present, especially at the level of individual diagnosis codes, whereas variations at the level of chapters and categories (symptom versus disease) are generally smaller.^18^ As the present data reflects Finnish primary health care, the setting itself is selective. We do not know the extent to which the results may apply to other primary health care settings.

### Comparison with existing literature

The prevalence of symptomatic diagnoses being 13.5% is in line with a previous study^9^ and with statistics provided by the Finnish register AvoHilmo. According to data from a Danish study of primary health care patients, about one third of the patients are judged to have a symptom without a specific diagnosis of disease.^15,19^ The main difference between the Danish study and ours was that we used the chapter R of the ICD-10 classification, whereas the Danish groups used the International Classification of Primary Care (ICPC) to record symptoms. There are some differences in recording health problems between these two classification systems. For example, acute stress reaction, which is very common in patients aged 18–65 years,^15^ is included in symptom-based diagnoses in ICPC, whereas in ICD-10, acute stress reaction is classified in psychiatric disorders (category F43). Furthermore, back and lower limb symptoms were considered as symptom-based diagnoses in the Danish study,^15^ whereas in the ICD-10 system they have diagnostic categories or subcategories of their own (M54 and M79.6). In line with the Danish study,^15^ we also observed that the quality of symptoms varied as a function of age.

An English study, focusing on the incidence of and factors predicting ill-defined (R-coded) hospital admissions of older people and their association with health outcomes, reported that the incidence of R-codes at discharge was 22%.^20^ In this study, the five most common symptomatic diagnoses were circulatory (including chest pain, syncope, and collapse) (28%), respiratory (28%), senility (14%), abdominal pain (11%), and cognitive symptoms (6%), which accounted for a total of 87% of the R-coded admissions. A Danish study found that R-coded diagnoses, together with Z-coded diagnoses, were frequent among patients hospitalised after calling the emergency number.^21^ According to the study, a non-specific diagnosis code (including both R and Z prefixes) constituted one-third of the hospital diagnoses assigned to the hospitalised patients. However, the hospital context is different from that of outpatient primary care. While reaching a specific diagnosis in urgent situations is essential, GPs often use time as a diagnostic tool in less severe cases.

### Implications for research

To the best of our knowledge, this is the first study to explore how age modifies the prevalence of symptomatic diagnoses. It is logical that general symptoms and signs as well as circulatory and respiratory symptoms increase with age, since multimorbidity and vascular diseases become more common as people age. However, it is interesting that the peak of symptoms in the digestive system and abdomen occurs in young adulthood. Otherwise, the lowest prevalence of symptoms is among middle-aged people.

Age is not the only factor which may be associated with the symptoms expressed. In the present data, the percentage of recorded symptom-based diagnoses was 14.1% among women and 12.4% among men. Therefore, sex may also influence what symptoms these patients complain of, as has been suggested before.^12,22^ As women live longer than men, they tend to experience greater levels of comorbidity and disability. Women can therefore be considered more frail because they have poorer health status, but more diseases that are not fatal. Globally, this is known as the ageing paradox.^23^ To our best knowledge, the present data are the first of their kind comparing sex differences of recorded symptom-based diagnoses according to age in primary health care. Our data suggest that, among the major symptom-based diagnoses, abdominal and pelvic pain and general symptoms are more predominant among women, while circulatory and skin-related symptoms are more common among men over the age of 60 years.

The most significant finding of this study is that patients report numerous non-specific symptoms to their GP, to which a definitive diagnosis cannot be assigned. This indicates that a tolerance of uncertainty is crucial in GPs’ work.^24^ Furthermore, follow up and knowing the patients are essential tools in GPs’ diagnostics. Continuity of care is an important aspect in quality of primary care and patient safety.^25^ Complaints based on symptoms that are experienced are also important from a clinical point of view because if a primary health care patient exhibits several symptoms, even with a specific disease, the risk of a premature disability pension increases.^26^ Therefore, it is not surprising that expressing several different symptoms, without any specific disease, has been suggested to constitute a diagnosis group of its own.^27^ A biopsychosocial explanation has been suggested to be more useful than a biomedical one in a therapeutic sense, especially in primary health care, for these multi-symptomatic patients.^28^ Mental stress factors, especially in middle age, may also play a considerable role in the expression of symptoms.^15,29^ Therefore, it is not always easy to judge whether or not expressing multiple symptoms without evident disease is associated with psychiatric disorders, such as anxiety,^4^ depression,^30^ and somatoform disorders.^31^

About one in eight diagnoses in primary health care is a symptomatic diagnosis, indicating uncertainty of the underlying disease. There are significant sex differences in prevalence within and between diagnosis groups of symptom-based diagnoses, and age is a major contributor for the diagnoses. The major symptom-based diagnoses of abdominal and pelvic pain, and general symptoms are more predominant among women while circulatory and skin-related symptoms are more common among men over the age of 60 years.

## Data

The dataset relied on in this article is available from the corresponding author on reasonable request.
